# Efficacy of Repetitive Transcranial Magnetic Stimulation in Patients With Methamphetamine Use Disorder: A Systematic Review and Meta-Analysis of Double-Blind Randomized Controlled Trials

**DOI:** 10.3389/fpsyt.2022.904252

**Published:** 2022-05-27

**Authors:** Chun-Hung Chang, Meng-Fen Liou, Chieh-Yu Liu, Wei-Hsin Lu, Shaw-Ji Chen

**Affiliations:** ^1^An Nan Hospital, China Medical University, Tainan City, Taiwan; ^2^Institute of Clinical Medical Science, China Medical University, Taichung City, Taiwan; ^3^Department of Psychiatry and Brain Disease Research Center, China Medical University Hospital, Taichung City, Taiwan; ^4^National Taipei University of Nursing and Health Sciences, Taipei, Taiwan; ^5^Biostatistical Consulting Lab, National Taipei University of Nursing and Health Sciences, Taipei, Taiwan; ^6^Department of Psychiatry, Ditmanson Medical Foundation Chia-Yi Christian Hospital, Chia-Yi City, Taiwan; ^7^Department of Psychiatry, Taitung MacKay Memorial Hospital, Taitung, Taiwan; ^8^Department of Medicine, Mackay Medical College, New Taipei City, Taiwan

**Keywords:** repetitive transcranial magnetic stimulation (rTMS), methamphetamine, craving, theta-burst stimulation, substance use disorder

## Abstract

**Background:**

Repetitive transcranial magnetic stimulation (rTMS) has demonstrated therapeutic potential for treating patients with methamphetamine use disorder (MUD). However, the most effective target and stimulation frequency of rTMS for treating MUD remains unclear. This meta-analysis explored the effect of rTMS on MUD.

**Methods:**

In this study, PubMed, Cochrane Systematic Reviews, and the Cochrane Collaboration Central Register of Controlled Clinical Trials were searched electronically for double-blind randomized controlled trials that used rTMS for treating MUD. We used published trials to investigate the efficacy of rTMS in MUD up to March 5, 2022, and pooled studies using a random-effect model to compare rTMS treatment effects. Patients who were diagnosed with MUD according to the criteria of the Diagnostic and Statistical Manual of Mental Disorders were recruited. Clinical craving scores between baseline and after rTMS were compared using the standardized mean difference (SMD) with 95% confidence intervals (CIs). The heterogeneity of the included trials was evaluated through a visual inspection of funnel plots and the I^2^ statistic.

**Results:**

We identified seven trials with 462 participants with MUD that met the inclusion criteria. All the studies evaluated craving scores, with rTMS demonstrating a more significant effect than the sham treatment on reducing craving scores (SMD = 0.983, CI = 0.620–1.345, *p* ≤ 0.001). A subgroup meta-analysis revealed that intermittent theta-burst stimulation (iTBS) had a greater positive effect than 10-Hz rTMS. A metaregression revealed that the SMDs increased with the increase in baseline craving scores, whereas they decreased with the increase in the proportion of men and duration of abstinence.

**Conclusion:**

The meta-analysis suggests that rTMS may be associated with treatment effect on craving symptoms in patients with MUD. iTBS may have a greater positive effect on craving reduction than 10-z rTMS.

## Introduction

Methamphetamine is a synthetic drug in Germany in 1887 and used widely during WWII by the Nazi and Japanese armies. Methamphetamine is medically used for the treatment of attention deficit hyperactive disorder and obesity (1). Methamphetamine is marketed as Desoxyn and Adderall in United States and other countries. Initially, students and young workers abuse methamphetamine because it could improve their performance by last their study and working time. However, methamphetamine is a highly addictive substance due to the tolerance of methamphetamine developing fast.

Methamphetamine initially improves a person’s awareness, focus, and physical performance, providing a feeling of euphoria. Additionally, its use leads to psychotic symptoms, such as anxiety, agitation, paranoia, and hallucinations. However, somatic symptoms are frequently experienced, such as seizures, chest pains, sweating, shortness of breath, palpitations, and high blood pressure.

The long-term use of methamphetamine usually results in a high dose because tolerance to the drug develops relatively rapidly. It may also trigger serious outcomes, such as arrhythmia and cerebral hemorrhage. The habitual use of methamphetamine often causes weight loss, poor cognitive functioning, persistent psychotic symptoms (e.g., persecutory delusions and hallucinations), and decreased sleep (2, 3).

The highest prevalence of methamphetamine abuse has been recorded in Asia, particularly in East and Southeast Asia, and this abuse is becoming a considerable socioeconomic burden worldwide according to the World Drug Report 2016 published by the United Nations Office on Drugs and Crime (UNODC). The UNODC estimates that 35.65 million people or 0.8% of the world’s population aged 15–64 was using methamphetamine in 2014.

People who abuse methamphetamine via different routes such as mouth ingestion, nose inhalation, or intravenous injection in different area of the world (4). Methamphetamine enter bloodstream rapidly after traverses the blood–brain barrier directly, entering the brain parenchyma because it is lipophilic. The drug mainly influences the reuptake of monoamine neurotransmitters such as dopamine, norepinephrine, and serotonin (1), increasing dopamine levels in the cytoplasm and neuromuscular junction. The abundance of dopamine provides the feeling of euphoria, explaining why chronic methamphetamine users feel unwell during withdrawal when their dopamine levels are low; hence, they feel the need for an increasing amount of stimulation (5).

Non-invasive brain stimulation including repetitive transcranial magnetic stimulation (rTMS) and transcranial direct current stimulation (tDCS) have been widely applied to different neurological and psychiatric conditions. It is considered to have therapeutic effects because of the neuromodulation produced by a change in unidentified mechanisms in the human brain that might include cortical excitability, neurotransmitter release, signaling pathways, and gene expression (6–10). Initially, rTMS was determined to have an antidepressant effect by inhibiting the dorsolateral prefrontal cortex (DLPFC). However, craving related to addiction is suspected to be correlated with the “brain reward circuit” through the dopamine pathway in the brain. Furthermore, inhibitory control is exerted by the DLPFC over the reward circuit through the mesofrontolimbic connections (11, 12). Studies have suggested that rTMS stimulates the DLPFC to reduce drug cravings through two processes. First, the DLPFC interacts with the ventral tegmental area, which is correlated with the reward system through an increase in dopamine. Second, stimulation of the DLPFC stimulates glutamate, inducing increased dopamine excretion and reducing cravings (13, 14). These findings support the use of rTMS for substance use disorders, although negative findings on alcohol and cocaine use disorder have also been revealed (15, 16). Therefore, in this study, we focused on methamphetamine use disorder (MUD), which is a central nervous system stimulant addiction similar to cocaine addiction. Studies on rTMS in relation to MUD have revealed that this treatment significantly reduces cravings and relapse (17–20). Theta-burst stimulation (TBS) is a novel TMS protocol in which short bursts of high-frequency (50 Hz) stimulation are repeated at 5 Hz (200-ms intervals). Both intermittent TBS (iTBS) and continuous TBS (cTBS) can rapidly induce synaptic plasticity (21). Pilot studies have reported the effects of TBS on patients with MUD (19, 22). However, the most effective frequency for both conventional rTMS and iTBS remains unclear. Because findings related to this promising anticraving intervention are varied, a meta-analysis of all studies on rTMS and MUD is warranted.

## Materials and Methods

### Search Strategy and Study Selection

**FIGURE 1:**
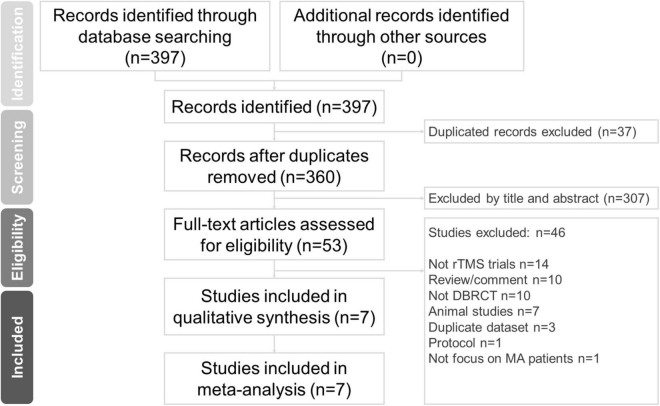
PRISMA flow diagram for the identification of included studies. Database: PubMed (*n* = 312), Cochrane Central Register of Controlled Trials (*n* = 85), Cochrane Database of Systematic Reviews (*n* = 0). Keyword: (methamphetamine OR methylamphetamine) AND (repetitive transcranial magnetic stimulation OR rTMS OR brain stimulation OR theta-burst). Date: date available to Mar 2022. DBRCT, double-blind randomized placebo-controlled trial; MA, methamphetamine; rTMS, repetitive transcranial magnetic stimulation.

In this study, two well-trained authors (C-HC and M-FL) independently performed a systematic literature search from the study’s inception until March 5, 2022. The search terms were (methamphetamine OR methylamphetamine) AND (repetitive transcranial magnetic stimulation OR rTMS OR brain stimulation OR theta-burst) (23–25). We searched the PubMed, Cochrane Collaboration Central Register of Controlled Clinical Trials, and Cochrane Systematic Reviews databases for studies on rTMS for MUD. The included trials and related review articles were reviewed manually to acquire pertinent references. The Preferred Reporting Items for Systematic Reviews and Meta-Analysis (PRISMA) guidelines were followed (26) (Figure 1).

### Eligibility Criteria

Studies were included if they (a) had participants with MUD, (b) were double-blind randomized placebo-controlled trials (DBRCTs), and (c) used rTMS as a monotherapy or adjunctive treatment. Articles were excluded if they (1) were not related to human clinical trials, (2) were review or comment papers, (3) did not include rTMS, (4) did not include a DBRCT, (5) were based on animal studies, (6) involved a duplicate dataset, (7) were a protocol, or (8) did not focus on patients with MUD.

### Data Extraction

The two authors independently extracted data of interest following the PRISMA guidelines. They examined all the retrieved articles and recorded information relating to the first author, year of publication, number of participants, sex ratios, mean age, baseline craving scores, brain target, frequency, number of sessions, onset age, duration of abstinence, duration of methamphetamine use, and methamphetamine dose per day (Table 1).

**TABLE 1:** Summary of the characteristics of the studies included in the meta-analysis.

Study (first author, year)	*N*	Gender (%male)	Mean age (years)	Baseline mean craving scores	Brain target	Frequency	Sessions	Onset age	Abstinence (months)	Duration of MA use (months)	MA (g/d)
Su et al. (20)	30	100.00	32.35 (5.03)	30.6 (31.54)	Left DLPFC	10 Hz	5	25.90 (5.21)	2.90 (1.63)	50.57 (37.02)	0.49 (0.33)
Liang et al. (28)	50	100.00	33.30 (9.80)	NA	Left DLPFC	10 Hz	10	NA	0.25 (0.14)	61.20 (37.85)	0.50 (0.30)
Su et al. (19)	126	84.10	31.66 (6.31)	46.52 (30.71)	Left DLPFC	iTBS	20	23.90 (6.79)	3.12 (1.66)	67.56 (41.40)	0.66 (0.43)
Yuan et al. (30)	73	100.00	38.49 (7.69)	22.63 (25.10)	Left DLPFC	1 Hz	10	NA	9.27 (4.61)	NA	NA
Chen et al. (17)	74	NA	34.89 (4.97)	45.18 (26.55)	Left DLPFC vmPFC	iTBS*	10	27.49 (5.64)	NA	73.56 (43.50)	NA
Su et al. (29)	60	63.20	32.62 (6.71)	29.72 (26.89)	Left DLPFC	iTBS	20	24.15 (7.48)	3.80 (1.24)	76.26 (44.34)	0.57 (0.34)
Chen et al. (22)	49	63.00	30.08 (5.54)	57.68 (30.80)	Left DLPFC	iTBS	20	23.54 (6.17)	2.79 (1.41)	60.00 (41.83)	NA

DLPFC, dorsolateral prefrontal cortex; iTBS, intermittent theta-burst stimulation; NA, not available; vmPFC, ventromedial prefrontal cortex. *Four-arm trial (Group A: iTBS targeting the left DLPFC; Group B: cTBS targeting the left vmPFC; Group C: a combination of the Group A and B treatment protocols; Group D: sham TBS).

### Methodological Quality Appraisal

In this study, Jadad scoring (27) was used to assess the methodological quality of the randomized controlled trials (RCTs) in the enrolled studies. Jadad scores evaluate the methodology quality of RCTs based on the following three aspects: (a) randomization (two points), (b) blinding (two points), and (c) an account of all patients (one point). Potential Jadad scores range from 0 to 5, with a higher score indicating higher methodological quality. Between-reviewer discrepancies were solved through discussions under the supervision of the corresponding author.

### Outcome Measures

We aimed to evaluate the rTMS effect on craving in participants with MUD. In this study, the reduced craving scores for rTMS and sham treatment were compared.

### Data Synthesis and Analysis

We used the standardized mean difference (SMD), which expresses changes in craving scores, in each selected meta-analysis to calculate the SMD. Positive values indicated that the craving scores improved after rTMS or sham therapy. We used a random-effects model to pool the individual SMDs. We used I^2^ tests to evaluate between-trial heterogeneity, and values > 50% were considered to indicate considerable heterogeneity. Two-tailed *p* values of < 0.05 were considered statistically significant. We used a sensitivity test with a “one study removal” test to evaluate the effect on the results of removing each individual study and reanalyzing the overall effect on the remaining studies. In addition, we evaluated potential publication bias with funnel plots and an Egger’s test. The meta-analysis was performed using Comprehensive Meta-Analysis software, version 3 (Biostat, Englewood, NJ, United States).

## Results

### Characteristics of Included Studies

The seven included studies had enrolled a total of 462 patients with MUD (mean age = 33.44 ± 2.57 years, men = 84.44%). The average number of participants was 78.10 ± 31.67 (range: 20–65), and the average number of treatment sessions was 14.76 ± 5.47 (range: 5–20). The mean baseline craving scores were 39.30 ± 10.87. The mean age of onset was 24.86 ± 1.52 years, and the mean duration of methamphetamine use was 66.96 ± 7.28 months. Six trials (19, 20, 22, 28–30) were two-arm trials with a sham-controlled design, and one (17) was a four-arm trial (Group A: iTBS targeting the left DLPFC; Group B: cTBS targeting the left vmPFC; Group C: a combination of the Group A and B treatment protocols; Group D: sham TBS). A schematic of the search process is presented in Figure 1 and Table 1 summarizes the study characteristics.

## Overall Repetitive Transcranial Magnetic Stimulation Effect on Craving Analyses

### Meta-Analyses of Repetitive Transcranial Magnetic Stimulation Effect

**FIGURE 2:**
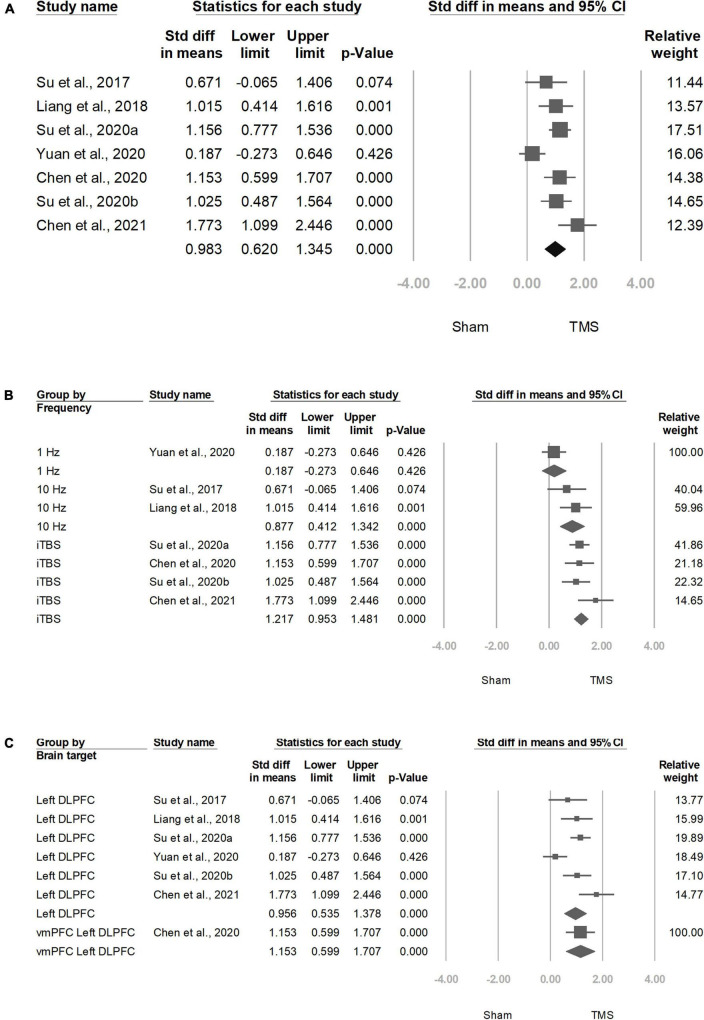
Meta-analyses of **(A)** overall standardized mean difference, **(B)** group by frequency, and **(C)** group by brain target.

Among the seven trials (17, 19, 20, 22, 28–30), all reported the effect on craving. The positive SMD results indicated the improvement of clinical symptoms after the treatment with add-on rTMS. rTMS showed a more significant effect than the sham treatment on reducing craving scores in participants with methamphetamine use disorder (SMD = 0.983, CI = 0.620–1.345, *P* ≤ 0.001; Figure 2A).

### Subgroup Analyses of Repetitive Transcranial Magnetic Stimulation Frequency

Four trials (17, 19, 22, 29) that used iTBS had significant ESs: 1.217 (95% CI: 0.953–1.481, *P* < 0.001), whereas two trials used 10 Hz showed significant ESs: 0.877 (95% CI: 00.412–1.342, *P* < 0.001; Figure 2B).

### Subgroup Analyses of Brain Target

Six studies (19, 20, 22, 28–30) targeted at left DLPFC showed significant ESs: 0.956 (95% CI: 0.535–1.378, *P* < 0.001; Figure 2C).

### Meta-Regression Analyses of Overall Clinical Symptoms

**FIGURE 3:**
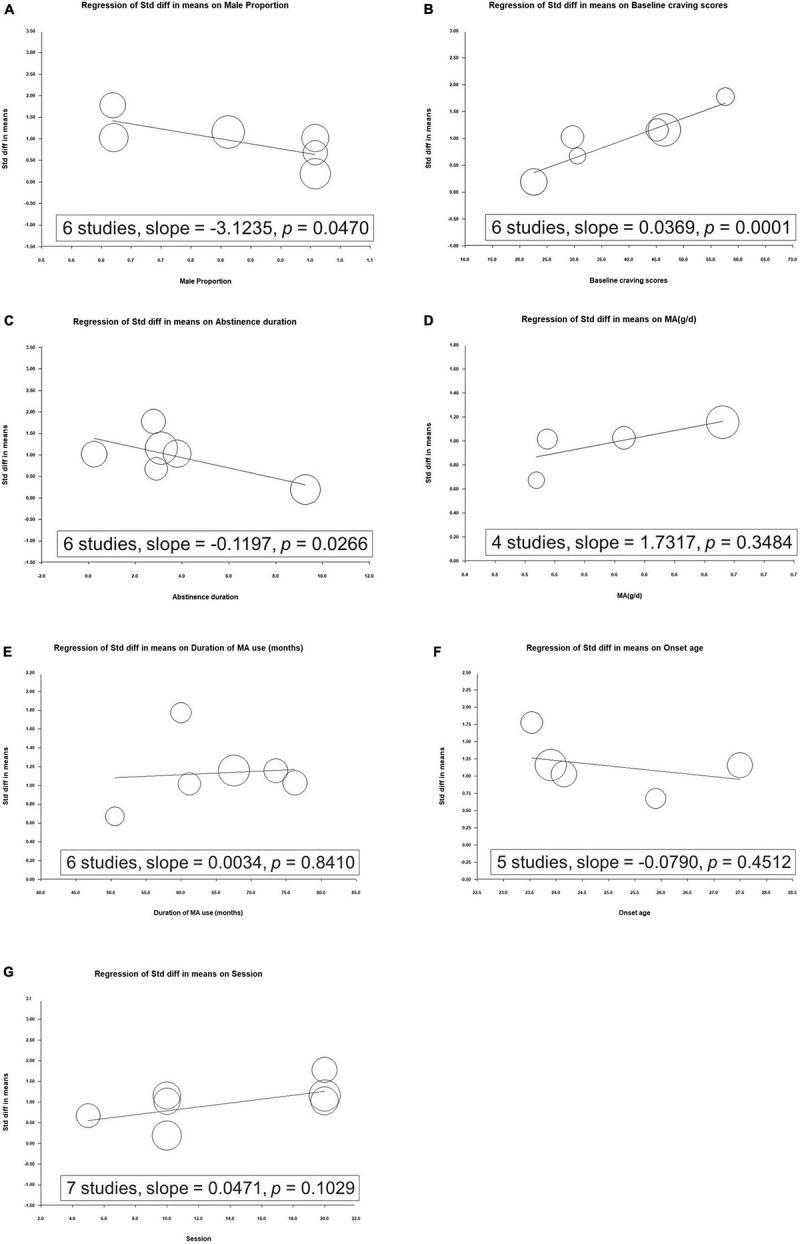
Metaregression of the effects of repetitive transcranial magnetic stimulation on craving in relation to **(A)** proportion of men, **(B)** baseline craving scores, **(C)** duration of abstinence, **(D)** mean methamphetamine use per day, **(E)** duration of methamphetamine use, **(F)** onset age, and **(G)** number of sessions.

We noted that the increased effect of rTMS on reducing craving scores was significantly correlated with the baseline craving scores, whereas a decreased effect of rTMS on craving scores was correlated with the proportion of men and duration of abstinence (Figure 3).

### Heterogeneity and Publication Bias

**FIGURE 4:**
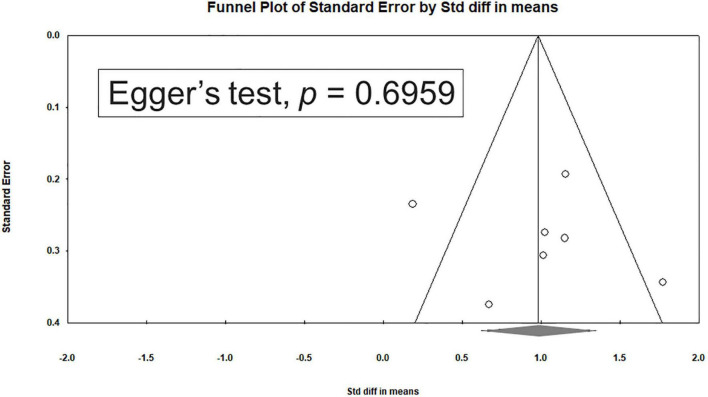
Funnel plots for the standardized mean differences of craving.

Significant heterogeneity was observed within the seven studies (*Q* = 18.641, df = 6, I^2^ = 67.814%, *P* = 0.005). Egger’s test revealed no significant publication bias regarding the overall SMD (*P* = 0.6959). The funnel plots for the SMD of overall clinical symptoms are shown in Figure 4.

### Sensitivity Analysis

In the meta-analysis of the rTMS effect on reducing craving scores, the conclusion remained significant when removing any single study.

## Discussion

To the best of our knowledge, this is the first meta-analysis focusing on the efficacy of rTMS in patients with MUD. We revealed that (1) rTMS had a significant positive effect on craving score reduction in participants with MUD compared with a sham treatment (SMD = 0.983, CI = 0.620–1.345, *p* < 0.001), (2) studies targeting the left DLPFC revealed significant positive effects, (3) TBS had a greater positive effect than 10-Hz rTMS, and (4) the ES increased with the baseline craving scores and decreased with the proportion of male participants and duration of abstinence.

Our findings are consistent with those of three meta-analyses (23–25). We included seven DBRCTs in our study, whereas other studies had four (25), five (24) and six (23). Zhang et al. included 26 trials, with four (20, 28, 31, 32) focusing on MUD. The ESs of these four trials ranged from a Hedges’ *g* of −0.398 to −1.611. Ma et al. included 12 trials, with five (20, 28, 31–33) focusing on rTMS and MUD. The mixed-effect subgroup analysis suggested that the treatment for methamphetamine addiction was positive (*N* = 10, Hedges’ *g* = 1.541, CI = [0.735, 2.347], *z* = 3.749, *p* < 0.001). Gay et al. included 34 trials, with six (19, 20, 28, 30, 31, 33) focusing on MUD. The subgroup analysis using a random-effects model demonstrated a significant positive effect on reducing methamphetamine cravings (SMD = −0.57, CI = −0.96 to −0.18, *z* = 2.83, *p* = 0.005). These meta-analyses reveal the effect of rTMS on methamphetamine cravings. In our study, the overall effect on craving was SMD = 0.983, CI = 0.620–1.345, *p* < 0.001. The differences in effect results might be explained by design of the included trials and ES methodology.

Moreover, in our study, high-frequency rTMS, including 10-Hz rTMS and TBS, had a significant effect on craving reduction, whereas low-frequency rTMS did not (Figure 2B). Our findings are consistent with other meta-analyses on substance use disorders. A systematic meta-analysis of non-invasive brain stimulation on stimulant-craving users of cocaine, amphetamine, and methamphetamine reported that in studies using high-frequency rTMS (*N* = 7), the craving level decreased (Hedges’ *g* = 1.671, CI = [0.669, 2.673], *z* = 3.269, *p* = 0.001), but in low-frequency rTMS studies, it did not (*N* = 4, Hedges’ *g* = 0.962, CI = [−1.137, 3.061], *z* = 0.898, *p* = 0.369) (24). Another meta-analysis of the effect of rTMS on craving in patients with substance dependence reported that in studies using excitatory rTMS over the left DLPFC (*N* = 13), the craving level decreased (Hedges’ *g* = −0.624, CI = [−0.894, −0.354], *z* = −4.531, *p* < 0.0001) (25). A single-blind sham-controlled crossover study enrolled 10 non-treatment-seeking methamphetamine-dependent users and discovered that low-frequency (1 Hz) rTMS of the left DLPFC transiently increased cue-induced craving for methamphetamine (1-Hz rTMS group: 17.86 ± 1.46 vs. sham group: 24.85 ± 1.57, *p* = 0.001) (31). Another study enrolled 50 male methamphetamine users and randomly assigned them to five groups (10 Hz left P3, 10 Hz L-DLPFC, 10 Hz R-DLPFC, 1 Hz L-DLPFC, 1 Hz R-DLPFC), revealing that on either the left or right side, both high-frequency and low-frequency rTMS were effective at decreasing the cue-induced cravings (32). However, this study lacked a sham control and was not double blinded.

We further evaluated different levels of high frequency. Two articles (20, 28) included in our meta-analysis investigated the effects of 10-Hz rTMS, whereas four studies (17, 19, 22, 29) investigated TBS. We noted that iTBS treatment was more effective than the 10-Hz treatment (*N* = 4, SMD = 1.217, *p* < 0.001 and *N* = 2, SMD = 0.877, *p* < 0.001, respectively). We aimed to clarify why patients receiving TBS experienced greater benefits than those receiving conventional 10-Hz rTMS. A network meta-analysis of the acute treatment of major depression enrolled 81 studies. Their results indicated that TBS is more effective than high-frequency rTMS in terms of remission (TBS: odds ratio [OR] = 3.37, 95% CI = 0.52–22.05; high-frequency rTMS: OR = 2.73, 95% CI = 1.78–4.20) (34). A randomized multicenter non-inferiority clinical trial evaluated the effectiveness of theta-burst versus high-frequency rTMS in patients with depression (35). They randomly allocated 205 participants to 10-Hz rTMS treatment and 209 to iTBS treatment. After 4–6 weeks of treatment, Hamilton rating scale for depression (17-item version) scores improved from 23.5 (SD 4.4) to 13.4 (7.8) in the 10-Hz rTMS group and from 23.6 (4.3) to 13.4 (7.9) in the iTBS group (adjusted difference = 0.103 [corrected], 95% CI = −1.16, *p* = 0.0011), indicating the non-inferiority of iTBS. Notably, these studies focused on depression rather than MUD. Further studies are warranted to evaluate the difference between TBS and high-frequency rTMS in patients with MUD.

We further evaluated the effect of each modulator on craving reduction. Through a metaregression analysis, we identified trials with a higher proportion of men demonstrating lower SMDs for the effects of rTMS on craving (Figure 2A). Previous studies have observed potential sex-related differences in rTMS-induced cortical plasticity. Inghilleri et al. observed that the motor-evoked potential (MEP) size increased progressively during women’s menstrual cycle, suggesting that rTMS may induce increased MEP in women in the late stage of the menstrual cycle (36). This is consistent with the results of other studies, highlighting the excitatory neuronal effect associated with estradiol and inhibition associated with progesterone (37, 38). A meta-analysis of rTMS used to treat patients with major depression observed that women may have a greater response to rTMS treatment than men (39). However, whether rTMS treatment yields a comparable sex-related difference in methamphetamine abuse populations remains undetermined. Further well-designed studies with larger samples are required to evaluate sex-related differences in relation to treatment and brain function.

In the metaregression analysis, we revealed that the increased effect of rTMS on craving scores was significantly correlated with baseline craving scores, whereas a decrease in the effect of rTMS on craving scores was correlated with the duration of abstinence (Figures 3B,C). Studies have noted that methamphetamine withdrawal may cause long-term effects, including dry mouth, paranoia, itching, sleeplessness, psychosis, and depressive symptoms (40). Craving is associated with withdrawal discomfort during abstinence (41, 42); thus, in the early stages of withdrawal, rTMS may have a greater effect on craving scores. Further studies should evaluate the different stages of abstinence after discontinuing methamphetamine use.

### Strengths and Implication

Our study has several strengths compared with the three other meta-analyses (23–25). First, we included seven trials, whereas other studies included four, five, and six (23–25). Second, we conducted a subgroup analysis on conventional rTMS and TBS, revealing that TBS had a higher positive effect on cravings than 10-Hz rTMS. Third, we used a metaregression to analyze the relationship between ES and key factors. In addition to rTMS, non-invasive brain stimulation like tDCS have shown promising effect in substance use disorder (10). A randomized and sham-controlled trial including 60 male patients showed that the combination of Matrix Model psychotherapy and tDCS may improve cognition and craving in MUD (9). Further trials are suggested to evaluate the treatment effect of rTMS and tDCS in patients with MUD.

### Limitations

Our study also has some limitations. First, the numbers of included trials and patients were small. Second, the duration of most trials was less than 36 weeks, and the long-term positive effect of rTMS treatment on craving remains uncertain. Third, not all trials used the same protocols to evaluate craving and rTMS treatment. Fourth, we did not consider trials without a double-blind design or unpublished studies. Five, comprehensive genetic or psychosocial factors that are potential confounders of treatment outcomes were not evaluated in this study. Further trials with larger sample sizes and including comprehensive variables may be warranted.

## Conclusion

This meta-analysis revealed that rTMS has a significantly positive effect on patients with MUD and a positive effect on craving reduction. In addition, iTBS has a greater positive effect on craving reduction than 10-*z* rTMS, and the effect correlated with an increased proportion of women. Further trials with larger sample sizes are suggested to evaluate these findings and explore the role of rTMS in patients with MUD.

## Data Availability Statement

The original contributions presented in the study are included in the article/supplementary material, further inquiries can be directed to the corresponding author.

## Author Contributions

C-HC and M-FL drafted the initial manuscript. C-YL and W-HL provided suggestions and reviewed the manuscript. S-JC critically reviewed the draft of manuscript, and approved the final submitted version manuscript. All authors contributed to the article and approved the submitted version.

## Conflict of Interest

The authors declare that the research was conducted in the absence of any commercial or financial relationships that could be construed as a potential conflict of interest.

## Publisher’s Note

All claims expressed in this article are solely those of the authors and do not necessarily represent those of their affiliated organizations, or those of the publisher, the editors and the reviewers. Any product that may be evaluated in this article, or claim that may be made by its manufacturer, is not guaranteed or endorsed by the publisher.

## References

[1] Kish SJ. Pharmacologic mechanisms of crystal meth. *Can Med Assoc J.* (2008) 178:1679–82. 10.1503/cmaj.071675 18559805 PMC2413312

[2] Freckelton I. Methamphetamine-induced psychosis and mental impairment: a challenge from New Zealand. *J Law Med.* (2019) 27:284–93. 32129036

[3] Martinotti G De Risio L Vannini C Schifano F Pettorruso M Di Giannantonio M. Substance-related exogenous psychosis: a postmodern syndrome. *CNS Spectr.* (2021) 26:84–91. 10.1017/s1092852920001479 32580808

[4] Mc KR Quinn B Higgs P Berk M Dean OM Turner A Clinical and demographic characteristics of people who smoke versus inject crystalline methamphetamine in Australia: findings from a pharmacotherapy trial. *Drug Alcohol Rev.* (2021) 40:1249–55. 10.1111/dar.13183 33022140

[5] Karila L Weinstein A Aubin H-J Benyamina A Reynaud M Batki SL. Pharmacological approaches to methamphetamine dependence: a focused review. *Br J Clin Pharmacol.* (2010) 69:578–92. 10.1111/j.1365-2125.2010.03639.x 20565449 PMC2883750

[6] George MS Lisanby SH Sackeim HA. Transcranial magnetic stimulation: applications in neuropsychiatry. *Arch Gen Psychiatry.* (1999) 56:300–11. 10.1001/archpsyc.56.4.300 10197824

[7] Post A Keck ME. Transcranial magnetic stimulation as a therapeutic tool in psychiatry: what do we know about the neurobiological mechanisms? *J Psychiatr Res.* (2001) 35:193–215. 10.1016/S0022-3956(01)00023-1 11578638

[8] George MS Nahas Z Kozel FA Li X Denslow S Yamanaka K Mechanisms and state of the art of transcranial magnetic stimulation. *J ECT.* (2002) 18:170–81. 10.1097/00124509-200212000-00002 12468991

[9] Fayaz Feyzi Y Vahed N Sadeghamal Nikraftar N Arezoomandan R. Synergistic effect of combined transcranial direct current stimulation and matrix model on the reduction of methamphetamine craving and improvement of cognitive functioning: a randomized sham-controlled study. *Am J Drug Alcohol Abuse.* (2022):1–10. 10.1080/00952990.2021.2015771 35404725

[10] Lupi M Martinotti G Santacroce R Cinosi E Carlucci M Marini S Transcranial direct current stimulation in substance use disorders: a systematic review of scientific literature. *J ECT.* (2017) 33:203–9. 10.1097/YCT.0000000000000401 28272095

[11] Strafella AP Paus T Barrett J Dagher A. Repetitive transcranial magnetic stimulation of the human prefrontal cortex induces dopamine release in the caudate nucleus. *J Neurosci.* (2001) 21:Rc157. 10.1523/JNEUROSCI.21-15-j0003.2001 11459878 PMC6762641

[12] Sanna A Fattore L Badas P Corona G Diana M. The hypodopaminergic state ten years after: transcranial magnetic stimulation as a tool to test the dopamine hypothesis of drug addiction. *Curr Opin Pharmacol.* (2021) 56:61–7. 10.1016/j.coph.2020.11.001 33310457

[13] Amiaz R Levy D Vainiger D Grunhaus L Zangen A. Repeated high-frequency transcranial magnetic stimulation over the dorsolateral prefrontal cortex reduces cigarette craving and consumption. *Addiction.* (2009) 104:653–60. 10.1111/j.1360-0443.2008.02448.x 19183128

[14] Barr MS Farzan F Wing VC George TP Fitzgerald PB Daskalakis ZJ. Repetitive transcranial magnetic stimulation and drug addiction. *Int Rev Psychiatry.* (2011) 23:454–66. 10.3109/09540261.2011.618827 22200135

[15] Maiti R Mishra BR Hota D. Effect of high-frequency transcranial magnetic stimulation on craving in substance use disorder: a meta-analysis. *J Neuropsychiatry Clin Neurosci.* (2017) 29:160–71. 10.1176/appi.neuropsych.16040065 27707195

[16] Martinotti G Pettorruso M Montemitro C Spagnolo PA Acuti Martellucci C Di Carlo F Repetitive transcranial magnetic stimulation in treatment-seeking subjects with cocaine use disorder: a randomized, double-blind, sham-controlled trial. *Prog Neuropsychopharmacol Biol Psychiatry.* (2022) 116:110513. 10.1016/j.pnpbp.2022.110513 35074451

[17] Chen T Su H Li R Jiang H Li X Wu Q The exploration of optimized protocol for repetitive transcranial magnetic stimulation in the treatment of methamphetamine use disorder: a randomized sham-controlled study. *EBioMedicine.* (2020) 60:103027. 10.1016/j.ebiom.2020.103027 32980696 PMC7522737

[18] Chen T Su H Jiang H Li X Zhong N Du J Cognitive and emotional predictors of real versus sham repetitive transcranial magnetic stimulation treatment response in methamphetamine use disorder. *J Psychiatr Res.* (2020) 126:73–80. 10.1016/j.jpsychires.2020.05.007 32422456

[19] Su H Chen T Jiang H Zhong N Du J Xiao K Intermittent theta burst transcranial magnetic stimulation for methamphetamine addiction: a randomized clinical trial. *Eur Neuropsychopharmacol.* (2020a) 31:158–61. 10.1016/j.euroneuro.2019.12.114 31902567

[20] Su H Zhong N Gan H Wang J Han H Chen T High frequency repetitive transcranial magnetic stimulation of the left dorsolateral prefrontal cortex for methamphetamine use disorders: a randomised clinical trial. *Drug Alcohol Depend.* (2017) 175:84–91. 10.1016/j.drugalcdep.2017.01.037 28410525

[21] Huang YZ Edwards MJ Rounis E Bhatia KP Rothwell JC. Theta burst stimulation of the human motor cortex. *Neuron.* (2005) 45:201–6. 10.1016/j.neuron.2004.12.033 15664172

[22] Chen T Su H Wang L Li X Wu Q Zhong N Modulation of methamphetamine-related attention bias by intermittent theta-burst stimulation on left dorsolateral prefrontal cortex. *Front Cell Dev Biol.* (2021) 9:667476. 10.3389/fcell.2021.667476 34414178 PMC8370756

[23] Gay A Cabe J De Chazeron I Lambert C Defour M Bhoowabul V Repetitive transcranial magnetic stimulation (rTMS) as a promising treatment for craving in stimulant drugs and behavioral addiction: a meta-analysis. *J Clin Med.* (2022) 11:624. 10.3390/jcm11030624 35160085 PMC8836499

[24] Ma T Sun Y Ku Y. Effects of non-invasive brain stimulation on stimulant craving in users of cocaine, amphetamine, or methamphetamine: a systematic review and meta-analysis. *Front Neurosci.* (2019) 13:1095. 10.3389/fnins.2019.01095 31680830 PMC6813242

[25] Zhang JJQ Fong KNK Ouyang RG Siu AMH Kranz GS. Effects of repetitive transcranial magnetic stimulation (rTMS) on craving and substance consumption in patients with substance dependence: a systematic review and meta-analysis. *Addiction.* (2019) 114:2137–49. 10.1111/add.14753 31328353

[26] Moher D Liberati A Tetzlaff J Altman DG Group P. Preferred reporting items for systematic reviews and meta-analyses: the PRISMA statement. *J Clin Epidemiol.* (2009) 62:1006–12. 10.1016/j.jclinepi.2009.06.005 19631508

[27] Jadad AR Moore RA Carroll D Jenkinson C Reynolds DJ Gavaghan DJ Assessing the quality of reports of randomized clinical trials: is blinding necessary? *Control Clin Trials.* (1996) 17:1–12. 10.1016/0197-2456(95)00134-4 8721797

[28] Liang Y Wang L Yuan TF. Targeting withdrawal symptoms in men addicted to methamphetamine with transcranial magnetic stimulation: a randomized clinical trial. *JAMA Psychiatry.* (2018) 75:1199–201. 10.1001/jamapsychiatry.2018.2383 30208372 PMC6583874

[29] Su H Liu Y Yin D Chen T Li X Zhong N Neuroplastic changes in resting-state functional connectivity after rTMS intervention for methamphetamine craving. *Neuropharmacology.* (2020b) 175:108177. 10.1016/j.neuropharm.2020.108177 32505485

[30] Yuan J Liu W Liang Q Cao X Lucas MV Yuan TF. Effect of low-frequency repetitive transcranial magnetic stimulation on impulse inhibition in abstinent patients with methamphetamine addiction: a randomized clinical trial. *JAMA Netw Open.* (2020) 3:e200910. 10.1001/jamanetworkopen.2020.0910 32167568 PMC7070234

[31] Li X Malcolm RJ Huebner K Hanlon CA Taylor JJ Brady KT Low frequency repetitive transcranial magnetic stimulation of the left dorsolateral prefrontal cortex transiently increases cue-induced craving for methamphetamine: a preliminary study. *Drug Alcohol Depend.* (2013) 133:641–6. 10.1016/j.drugalcdep.2013.08.012 24028801 PMC4196687

[32] Liu Q Shen Y Cao X Li Y Chen Y Yang W Either at left or right, both high and low frequency rTMS of dorsolateral prefrontal cortex decreases cue induced craving for methamphetamine. *Am J Addict.* (2017) 26:776–9. 10.1111/ajad.12638 29134789

[33] Liu T Li Y Shen Y Liu X Yuan TF. Gender does not matter: add-on repetitive transcranial magnetic stimulation treatment for female methamphetamine dependents. *Prog Neuropsychopharmacol Biol Psychiatry.* (2019) 92:70–5. 10.1016/j.pnpbp.2018.12.018 30605708

[34] Brunoni AR Chaimani A Moffa AH Razza LB Gattaz WF Daskalakis ZJ Repetitive transcranial magnetic stimulation for the acute treatment of major depressive episodes: a systematic review with network meta-analysis. *JAMA Psychiatry.* (2017) 74:143–52. 10.1001/jamapsychiatry.2016.3644 28030740

[35] Blumberger DM Vila-Rodriguez F Thorpe KE Feffer K Noda Y Giacobbe P Effectiveness of theta burst versus high-frequency repetitive transcranial magnetic stimulation in patients with depression (THREE-D): a randomised non-inferiority trial. *Lancet.* (2018) 391:1683–92. 10.1016/S0140-6736(18)30295-2 29726344

[36] Inghilleri M Conte A Curra A Frasca V Lorenzano C Berardelli A. Ovarian hormones and cortical excitability. an rTMS study in humans. *Clin Neurophysiol.* (2004) 115:1063–8. 10.1016/j.clinph.2003.12.003 15066531

[37] Smith MJ Keel JC Greenberg BD Adams LF Schmidt PJ Rubinow DA Menstrual cycle effects on cortical excitability. *Neurology.* (1999) 53:2069–72. 10.1212/wnl.53.9.2069 10599783

[38] Smith MJ Adams LF Schmidt PJ Rubinow DR Wassermann EM. Effects of ovarian hormones on human cortical excitability. *Ann Neurol.* (2002) 51:599–603. 10.1002/ana.10180 12112106

[39] Kedzior KK Azorina V Reitz SK. More female patients and fewer stimuli per session are associated with the short-term antidepressant properties of repetitive transcranial magnetic stimulation (rTMS): a meta-analysis of 54 sham-controlled studies published between 1997-2013. *Neuropsychiatr Dis Treat.* (2014) 10:727–56. 10.2147/NDT.S58405 24855360 PMC4019615

[40] Clark M Featherstone R. *Management of Acute Withdrawal and Detoxification for Adults who Misuse Methamphetamine: A Review of the Clinical Evidence and Guidelines.* Ottawa (ON): Canadian Agency for Drugs and Technologies in Health (2019). 31411840

[41] Altshuler RD Lin H Li X. Neural mechanisms underlying incubation of methamphetamine craving: a mini-review. *Pharmacol Biochem Behav.* (2020) 199:173058. 10.1016/j.pbb.2020.173058 33250444 PMC7780755

[42] Paulus MP Stewart JL. Neurobiology, clinical presentation, and treatment of methamphetamine use disorder: a review. *JAMA Psychiatry.* (2020) 77:959–66. 10.1001/jamapsychiatry.2020.0246 32267484 PMC8098650

